# Mass Is All That Matters in the Size–Weight Illusion

**DOI:** 10.1371/journal.pone.0042518

**Published:** 2012-08-09

**Authors:** Myrthe A. Plaisier, Jeroen B. J. Smeets

**Affiliations:** 1 Cognitive Neurosciences Department, Bielefeld University, Bielefeld, Germany; 2 Faculty of Human Movement Sciences, VU University, Amsterdam, The Netherlands; Charité University Medicine Berlin, Germany

## Abstract

An object in outer space is weightless due to the absence of gravity, but astronauts can still judge whether one object is heavier than another one by accelerating the object. How heavy an object feels depends on the exploration mode: an object is perceived as heavier when holding it against the pull of gravity than when accelerating it. At the same time, perceiving an object’s size influences the percept: small objects feel heavier than large objects with the same mass (size–weight illusion). Does this effect depend on perception of the pull of gravity? To answer this question, objects were suspended from a long wire and participants were asked to push an object and rate its heaviness. This way the contribution of gravitational forces on the percept was minimised. Our results show that weight is not at all necessary for the illusion because the size–weight illusion occurred without perception of weight. The magnitude of the illusion was independent of whether inertial or gravitational forces were perceived. We conclude that the size–weight illusion does not depend on prior knowledge about *weights* of object, but instead on a more general knowledge about the *mass* of objects, independent of the contribution of gravity. Consequently, the size–weight illusion will have the same magnitude on Earth as it should have on the Moon or even under conditions of weightlessness.

## Introduction

The size-weight illusion is the well-known effect that large objects are perceived to be lighter than small objects of the same weight [1]. Although this illusion originally was discovered as a multi–sensory phenomenon in which the visually perceived size of an object influences its perceived weight, the illusion also occurs in the absence of vision if haptic size cues are available [2]. This shows that the illusion is not a multi-sensory one per se. It is based on a general effect of perceived size on perceived weight. In fact, showing an object before lifting, but not during lifting already triggers the illusion [3]. Similarly, there exists also a material–weight illusion [4], [5]: objects that are perceived to be made out of a denser material are perceived to be lighter than objects with the same size and weight that appear to be made out of a less dense material. Apparently, our percept of how heavy an object feels is biased by prior knowledge about the general relationship between object properties and the weight of an object. This suggests that the size–weight illusion occurs because we have learned that there is a correlation between size and weight. This idea is supported by a study in which it was shown that the illusion can be reversed: repeatedly lifting a set of objects manufactured such that the smaller objects had more mass than the larger objects for several days reduced the illusion and finally reversed it [6].

Combining information sources, such as size and weight, is common in human perception. It has been shown that when judging the size of an object through vision and touch simultaneously, the two estimates are integrated in a statistically optimal fashion [7]. This means that the combined percept is more precise than either of the two percepts independently. In fact, one can even learn to integrate two unrelated perceptual signals such as stiffness and luminance [8]. Sometimes, several information sources are combined with prior assumptions. This can be modelled using Bayesian statistics [9]. In the case of the size–weight illusion a perceptual estimate of size is combined with an estimate of the weight together with a prior for large objects being heavier. The way these information sources are combined in the size–weight illusion is fundamentally different from the previous examples, as it makes the percept less accurate and can be regarded as anti-Bayesian [10].

A prior for larger objects being heavier would suggest that a larger lifting force is applied for lifting large objects than for lifting small objects [11]. It has been shown that initially larger lifting forces are applied when lifting large objects, but the difference in the applied forces disappears within a few lifts while the perceived weight difference remains constant [12], [13]. This suggests that the illusion is not caused by applying more force when lifting a large object than when lifting a smaller object.

Since the illusion is usually referred to as size-weight illusion, one would expect it to be related to the weight of an object. Note that weight is another word for the gravitational force acting upon an object, which is proportional to the (gravitational) mass of an object. The mass of an object can also be experienced without weight through inertial forces proportional to the (inertial) mass acting during acceleration of an object [14]. This is why the mass of an object can be judged in the absence of gravity, such as in outer–space. Since gravitational and inertial masses of an object are the same (Einstein’s equivalence principle), one might expect that the two types of mass appear to be the same for the perceptual system. Surprisingly, an object is perceived to be almost twice as heavy through perception of gravitational pull than through perception of inertial forces [15], [16]. So, clearly the perceived heaviness of an object depends on whether inertial or gravitational forces are perceived, even though the underlying object property, mass, is the same.

In the present study we investigated whether the size-weight illusion depends on perceiving the pull of gravity, i.e. whether it is caused by a prior for weight or a by more general prior for the mass of an object. To this end we investigated whether the size–weight illusion occurs in the absence of weight through perception of inertial forces only. If the size–weight illusion occurs independent of gravitational forces, the illusion must be related to the mass of an object independent of the forces acting upon it. One reason for not expecting the illusion to occur in the absence of gravitational forces is that in daily life we rarely experience the heaviness of an object without perceiving gravitational forces. This means that the prior for larger objects being heavier may be limited to perception of gravitational forces, i.e. weight. Secondly, for perceiving inertial mass it is necessary to combine information about the acceleration of an object with efferent or afferent information about the applied amount of force. Gravitational forces (weight), in contrast, can be perceived purely tactual through the pressure of an object on the skin of the static hand. Therefore, fundamentally different sources of information are being used for perception of mass through inertial or gravitational forces.

To investigate whether the size–weight illusion occurs in the absence of weight, a set of objects differing in size but with the same mass was constructed. To let participants perceive inertial forces only, we suspended the objects from a long pair of wires and asked the participant to give the objects a short push after which they rated the perceived heaviness. This way perception of gravitational forces acting on the object was minimised. We let participants perform this task with and without visual feedback of the trajectory of the object after pushing to test whether participants used visual information about how far the object travelled. Finally, we also asked participants to rate heaviness after lifting the objects and placing them back as a control task. This allowed us to test whether the magnitude of the illusion as obtained through perception of inertial forces differed from the illusion obtained in the traditional way.

## Materials and Methods

### Participants

Twenty self-reported right-handed participants volunteered in the experiment (age range 22 to 40 years). All participants were naive as to the purpose of the experiment. Half of the participants performed the experiment pushing the objects with full vision. The other half also pushed the objects, but without visual feedback of the object’s trajectory. Eight of the subjects that performed the pushing with full visual feedback task also performed the control task of lifting with full vision.

### Ethics Statement

The experiment was conducted as part of a program that was approved by the ethical committee of the Faculty of Human Movement Sciences of VU University. All participants signed a statement of informed consent prior to participation in the experiments.

### Stimuli

**Figure 1:**
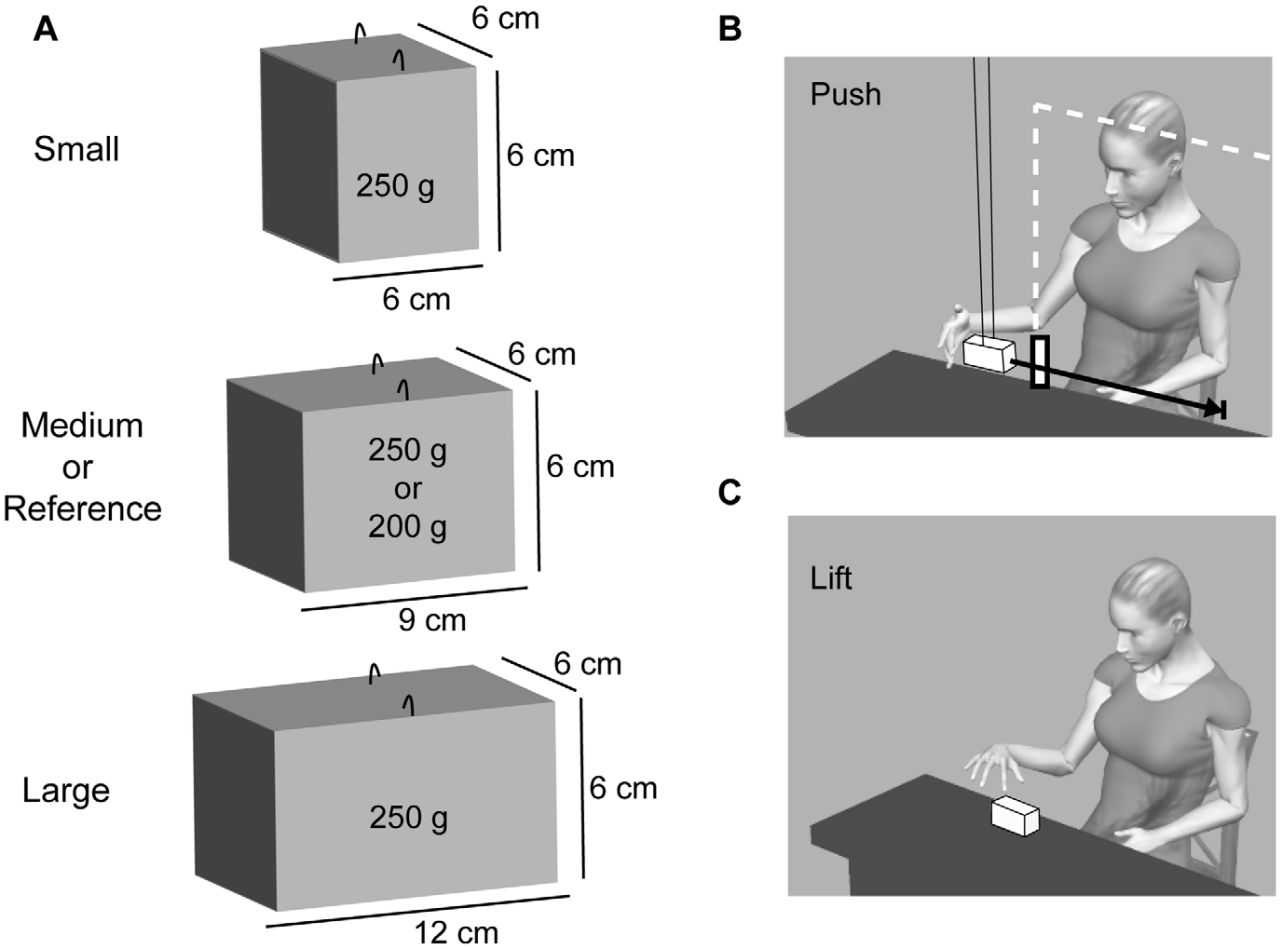
Description of the stimuli and set-up. (A) Dimensions of the four objects constructed out of medium density fibre (MDF). The three test objects (small, medium, large) had a mass of 250 g, the reference object had a mass of 200 g. Each object had two hooks attached to the top surface such that they could be suspended from a double wire attached to the ceiling. (B) A schematic representation of the set up. Subjects were seated in front of a table above which the objects could be suspended one at a time. The distance from the object to the ceiling (the effective length of the pendulum) was 2.3 m. The subjects were asked to push the object such that it travelled over an indicated distance of 50 cm (black arrow). Either an obstacle (withe bar; group with visual feedback) or a screen (white outline; group without visual feedback) ensured that the participant could not keep contact with the object for a distance larger than 10 cm. The experimenter caught and replaced the object after each push. (C) In the lifting task an object was placed on the table in front of the participant and he or she was asked to lift the object to a marked height (20 cm) and place it back on the table. The objects were always grasped on the horizontal 6 cm axes, such that grip aperture was the same for all object sizes.

The stimuli consisted of a set of four objects constructed out of MDF (medium density fibre). Three test objects differing in size (small, medium, large) were weighed down such that the mass of each object was 250 g (Fig. 1A). The fourth object (reference) had the same dimensions as the medium sized test object, but had a mass of 200 g. The ratings for the two medium sized objects were used to convert the heaviness ratings of the other objects into grams.

A small infrared Light Emitting Diode was attached in the centre of each object’s surface facing away from the subject. The position of the object was recorded at 500 Hz using an Optotrak position tracking system (Northern Digital). These data were used to calculate the objects’ velocities.

### Task

In the pushing task the objects were suspended just above table height from a pair of long wires in front of the participant. The participants were asked to give the object a short push such that it travelled over a distance of 50 cm (Fig. 1B) and rate how heavy the object felt using arbitrary numbers (i.e. method of absolute magnitude estimation [17]). These ratings were converted into z-scores by taking the difference between the individual ratings of a participant and his or her average rating, before dividing by the standard deviation of the ratings. For the task without visual feedback a screen was placed in front of the subject, such that the object was initially visual, but it disappeared behind the screen shortly after the push.

The procedure in the control task was the same as in the pushing task, but now participants were instructed to lift the objects between their thumb and index finger. They lifted the objects grasping them in the centre along the 6 cm axis, such that grip aperture was the same for all objects (Fig. 1C).

In all tasks the test objects were presented in 15 sets of three trials; in every set each object was presented once and the order of presentation within each set was randomised. After these 15 sets of three trials, another 10 trials were performed in which the reference object was presented 5 times randomly interleaved with 5 times one of the test objects. All trials were performed in one continuous run such that subjects were not aware of the introduction of the reference object.

### Analysis

Repeated measures ANOVA was performed on the *z*-scores of the heaviness ratings with object size as a repeated factor and visual feedback as a between subjects factor. The effect of object size was also tested with a repeated measures ANOVA on the peak velocities of the objects. Finally, a repeated measures ANOVA with object size and task as repeated factors was performed to compare the lifting and pushing with visual feedback conditions.

## Results

### Pushing

**Figure 2:**
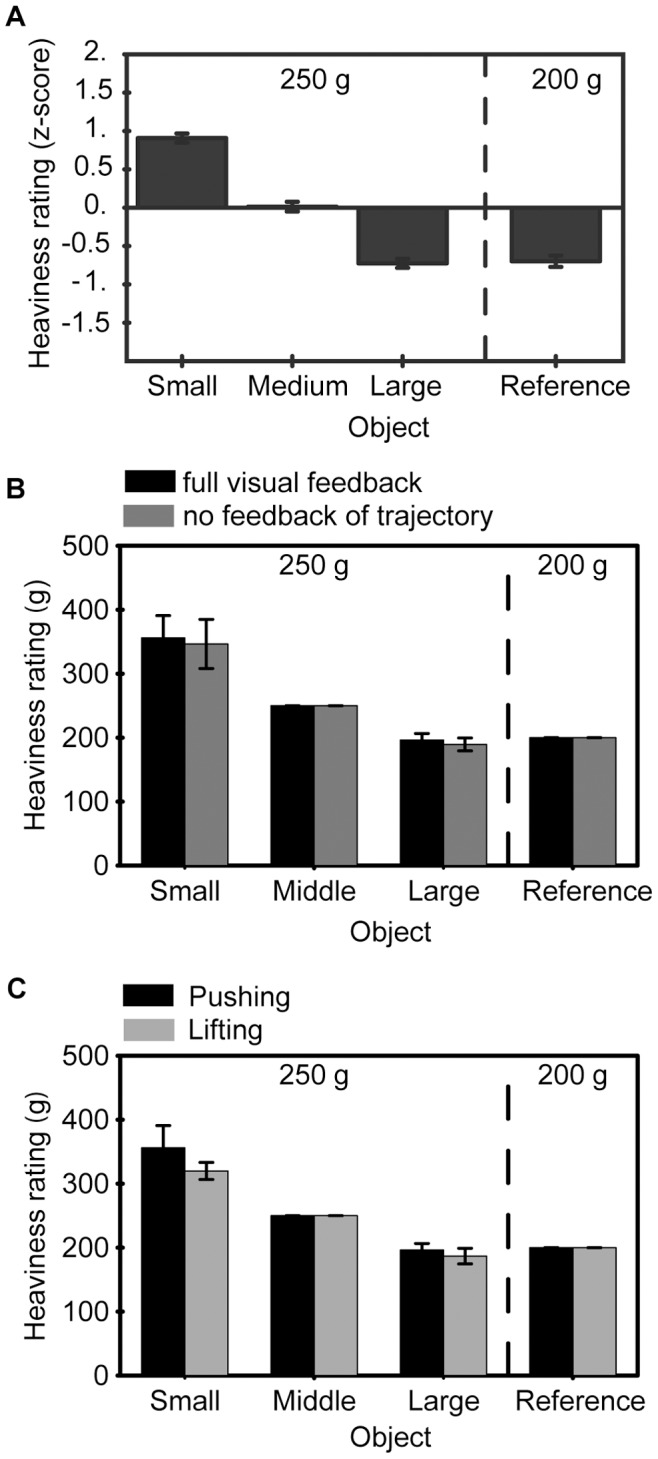
Heaviness ratings averaged over subjects. (A) Judged mass for pushing the objects with full visual feedback expressed as z-scores. (B) Judged mass expressed in grams for pushing with full visual feedback (black) and pushing without visual feedback of the trajectory (dark grey). The scores were normalised in such a way that the judgments for the medium and reference object corresponded to 250 g and 200 g, respectively, so that the standard error for these objects is zero. (C) Judged mass expressed in grams for subjects that performed both the pushing with full visual feedback (black) as well as the lifting (grey) task. In all panels the error bars represent the between–subjects standard error.

The heaviness ratings were converted into z-scores and are shown for the participants in the full vision tasks in Fig. 2A. Using the difference in the ratings for the two medium sized objects, the z-scores were converted into grams. The results show that the small 250 g object was consistently rated as being almost 100 g heavier than the large 250 g object. This was the case with full visual feedback and without vision of the object’s trajectory (Fig. 2B): a repeated measures ANOVA with object size as a within subjects factor and visual feedback as between subjects factor on the *z*-scores showed an effect of object size (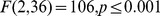) but no interaction between visual feedback and object size (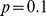). Therefore, the size–weight illusion occurs without any haptic or visual information about gravitational forces acting on the object. Furthermore, visual feedback of the trajectory did not affect the illusion size.

**Figure 3:**
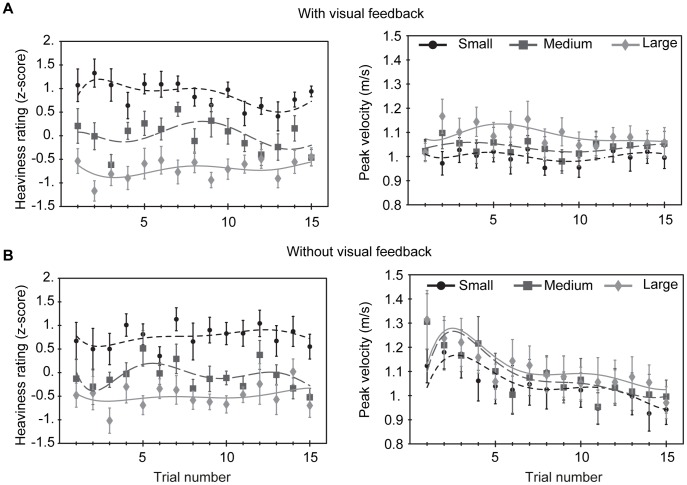
Heaviness ratings and peak velocities of the objects. Ratings and velocities are shown for each trial with (A) and without (B) visual feedback of the objects’ trajectories. The lines represent polynomials that were fitted to the data solely as a guide for the eye.

In Fig. 3A it can be seen that the peak velocity the object reached correlated with its size: the small object reached a lower peak velocity than the large object. This indicates that participants expected the small object to be lighter than the large one and therefore used less pushing force than for the large one resulting in a smaller peak velocity. Repeated measures ANOVA with object size as a within subjects factor on the peak velocities in the full visual feedback task showed an effect of object size (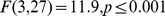). Participants that had no visual feedback of the trajectory of the object gradually reduced their force so that the peak velocity decreased over trials (Fig. 3B). Still, however, the peak velocity was larger for the small object than for the large object without visual feedback (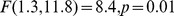, Greenhouse-Geisser corrected values). Although participants reduced their pushing force, the heaviness percept was stable over all trials. So different amounts of force were used for the different object sizes based on prior expectations, but the amount of force used itself was not a direct factor determining the percept.

### Lifting

After pushing, the small object was rated almost twice as heavy as the large object, which is a very large effect given that the volume was halved. The illusion found in our pushing task is larger than typically reported [2], [12]. We investigated whether this large magnitude of the illusion is due to our specific choice of objects or that gravity might reduce the magnitude of the size–mass illusion. To compare the magnitude of the illusion without gravity to the traditional size–weight illusion for our stimuli, we asked eight of the participants that performed the pushing with visual feedback to repeat the experiment but this time the task was to lift the objects (Fig. 1C). The judged heaviness is shown averaged over participants in Fig. 2C. It can be seen that the heaviness ratings are comparable between pushing and lifting. A 2×3 (task×object size) repeated measures ANOVA on the z-scores of the lifting and pushing with full visual feedback conditions showed only an effect of object size (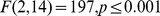). There was no effect of task nor was there an interaction effect (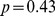 and 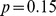, respectively).

## Discussion

First, we have shown that perception of weight is not at all necessary for the size–weight illusion to occur. The illusion should therefore be interpreted as a size–mass illusion. The results also show that the magnitude of the illusion was similar for lifting and pushing. This demonstrates that weight is not only unnecessary for the illusion to occur, but that also the magnitude of the illusion does not depend on providing weight as a cue. An explanation for the fact that the illusion size reported here is large compared to what has been reported previously is that we have used a set containing one cube, while the other objects had the same height and depth but were elongated compared to the cube. In other studies usually a set of cubes in different sizes was used. Possibly the visual estimate of the volume differences between the shapes was therefore more pronounced in our study. Anyway, the constancy of the magnitude of the illusion across lifting and pushing indicates that the size–mass illusion is independent of the basis of the heaviness percept.

Our results show that the objects with different sizes reached different peak velocities. There was no adaptation of the peak velocities over trials, i.e. participants didn’t adapt their pushing force such that all object sizes were pushed with the same amount of force after a number of trials. It has been shown that the maximum grip and load force rates adapt within as little as five trials such that there are no differences in these values anymore for lifting small and large objects [12]. This suggests that a mismatch between lifting force and an object’s weight is not an explanation for the size–weight illusion. It has, however, also been shown that the grip and load forces themselves do not adapt or at least do not adapt as fast as the peak grip and load force rates [18]. Since the peak velocity of the objects in our study is directly coupled to the amount of force applied (and not to its rate), adaptation is not to be expected for the peak velocities.

Generally, the size-weight illusion is explained in terms of a discrepancy between prior expectations and sensory information [6], [10]. This means that the perceptual system uses knowledge from prior experiences such as that larger objects are heavier than smaller ones [6]. Therefore different forces are used to lift small and large objects with the same mass [11], [12]. In daily life situations we perceive either only gravitational forces when statically holding an object or a combination of gravitational and inertial forces while moving an object, but we rarely experience inertial forces alone. Nonetheless, we found that prior experience handling objects results in the same illusion magnitude for lifting and pushing of objects. This shows that the size–weight illusion is not caused by a perceptual prior for the lifting forces directly associated with weight, but by a more general prior related to the mass of objects instead.

Several researchers have used the size–weight illusion to investigate the mechanisms underlying heaviness perception [19], [20]. The present study demonstrates that the size–weight illusion is very robust, and is independent of the mechanism underlying the heaviness percept. We found that the magnitude of the illusion is the same for lifting and pushing whereas the heaviness percept (and the mechanism it is based on) differs between these modes of exploration. Apparently, there is a discrepancy, because both modes of exploration show a large difference in perceived heaviness, but not in the magnitude of the size–weight illusion. This indicates that one should be careful when drawing conclusion about heaviness perception in general from results obtained from studying size–weight illusion.

In the physical world gravitational and inertial mass are the same according to Einstein’s equivalence principle. Mass is, however, perceived differently through inertial forces than through gravitational forces [15]. An explanation is that the perceptual system treats gravitational and inertial mass differently and thereby violates the equivalence principle [16]. Here we have shown that this violation is limited as the size–weight illusion is based on a prior for mass, regardless whether mass is perceived through gravitation or inertia. Therefore, we conclude that the illusion is based on a prior for the mass of an object, not a prior for forces acting on the object.
